# Automatic Pixel-Level Crack Detection on Dam Surface Using Deep Convolutional Network

**DOI:** 10.3390/s20072069

**Published:** 2020-04-07

**Authors:** Chuncheng Feng, Hua Zhang, Haoran Wang, Shuang Wang, Yonglong Li

**Affiliations:** 1 School of Information Engineering, Southwest University of Science and Technology, Mianyang 621000, China; beidou_stars@163.com (C.F.); swustai@163.com (H.Z.); 2 State Key Laboratory of Hydroscience and Engineering, Tsinghua University, Beijing 100084, China; 3 Sichuan Energy Internet Research Institute, Tsinghua University, Chengdu 610000, China; wangshuang@tsinghua-eiri.org (S.W.); liyonglong@hotmail.com (Y.L.)

**Keywords:** crack detection, dam surface, UAV, pixel-level, deep convolutional network

## Abstract

Crack detection on dam surfaces is an important task for safe inspection of hydropower stations. More and more object detection methods based on deep learning are being applied to crack detection. However, most of the methods can only achieve the classification and rough location of cracks. Pixel-level crack detection can provide more intuitive and accurate detection results for dam health assessment. To realize pixel-level crack detection, a method of crack detection on dam surface (CDDS) using deep convolution network is proposed. First, we use an unmanned aerial vehicle (UAV) to collect dam surface images along a predetermined trajectory. Second, raw images are cropped. Then crack regions are manually labelled on cropped images to create the crack dataset, and the architecture of CDDS network is designed. Finally, the CDDS network is trained, validated and tested using the crack dataset. To validate the performance of the CDDS network, the predicted results are compared with ResNet152-based, SegNet, UNet and fully convolutional network (FCN). In terms of crack segmentation, the recall, precision, F-measure and IoU are 80.45%, 80.31%, 79.16%, and 66.76%. The results on test dataset show that the CDDS network has better performance for crack detection of dam surfaces.

## 1. Introduction

With the rapid development of water conservancy projects, to meet the needs of power generation, shipping and irrigation, lots of hydropower stations are built. The dam is an important hydraulic structure of hydropower stations. Cracks caused by structural deformation, earthquakes, water flow impact and other factors have potential safety hazards for the normal operation of dam. Therefore, regular crack detection plays a crucial part in the maintenance and operation of existing dams. According to the morphological and apparent features of cracks, deterioration, and the potential causes can be inferred, which provides reasonable guidance for structural health diagnosis [1]. The crack detection of traditional human-based visual inspection is inefficient, subjective, and time-consuming. Therefore, automatic and efficient crack detection is highly essential for a structural health assessment of dams.

Automatic crack detection methods based on computer vision have been widely studied. Most of these methods adopt image processing technology and machine learning algorithm which can detect some simple types of structural damage [2,3]. Liu et al. [4] presented a method for tunnel crack detection and recognition using features of crack intensity and the support vector machine algorithm. Sinha and Fieguth [5] proposed a statistical filter for detection of cracks in the pipes by a two-step approach. Nishikawa et al. [6] developed multi-sequential image filter for detecting crack using several simple image filters. Cha et al. [7] proposed a vision-based method for structural damage detection by Hough transform and support vector machine. Shi et al. [8] proposed an automatic road crack detection method based on random structured forests. Zalama et al. [9] presented Gabor filters for road crack detection using AdaBoost algorithm. Li et al. [10] developed a method for bridge crack detection by active contour model and greedy search-based support vector machine. Yamaguchi et al. [11] introduced a crack detection method based on percolation model and length criterion. The accuracy of crack detection was still potentially to be improved [12]. Hence, three kinds of improvements were put forward: increasing the adaptation of global transforms [13,14,15], devising crack specific filters [16], and combing multiple global and local detectors [17,18]. These methods extract features from images using hand-picked feature and then evaluate whether the extracted features indicate defect. However, the results of the above methods have been inevitably affected by subjective factors.

In recent years, convolutional neural networks (CNN) have made great progress in image classification and target recognition [19,20,21,22]. Crack detection methods based on CNN have shown better performance than traditional image processing technology and machine learning methods [23,24]. Li et al. proposed a new application scenario for applying YOLOv3 to crack detection on dam surfaces and share its effects [25]. Makantasis et al. [26] proposed a tunnel crack detection method using deep convolutional neural network and multi-Layer perceptron. In order to enhance accuracy of traditional crack detection methods, Nhat-Duc et al. [27] developed a crack detection method employing edge detection algorithms and CNN. Feng et al. [28] proposed a deep active learning system to maximize the performance of crack detection, difficult cases were labeled by human experts in the training epoch. Using UASs with self-navigation abilities and improving image-processing algorithms to provide results near real-time could revolutionize the bridge inspection industry by providing accurate, multi-use, autonomous three-dimensional models and damage identification [29].

Khaloo et al. [30] proposed that a combination of multiple drone platforms and multi-scale photogrammetry technology was used to create two comprehensive and high-resolution 3D point clouds of the dam and the surrounding environment. Dorafshan et al. [31] investigated the feasibility of using a Deep Learning Convolutional Neural Network (DLCNN) in inspection of concrete decks and buildings using small Unmanned Aerial Systems. Undeniably, these methods achieve excellent accuracy of crack classification, but crack locating is highly necessary for crack detection. The Region-Based deep learning method has been used for detecting cracks. Kim et al. [32] suggested a bridge crack detection method combining unmanned aerial vehicles and region with convolutional neural networks (R-CNN)-based transfer learning. Cha et al. [33] proposed a region proposal network method for damage detection based on Faster R-CNN framework [34]. Xue et al. [35] proposed a fully convolutional network method for shield tunnel lining defects using GoogLeNet and Faster R-CNN. Li et al. [36] designed a supervised deep convolutional neural network and proposed novel training methods to optimize its performance on simultaneous concrete defect detection. Extensive experiments showed that the proposed method is effective with a detection accuracy of 80.7% and a localization accuracy of 86% at 0.41 s per image. Chen et al. [37] developed a deep convolutional neural network combined with Naïve Bayes to detect crack patches of nuclear power plants. Although previous researchers have proposed highly accurate methods for automatic crack detection, the existing detection methods cannot be used to analyze the images of dams directly owing to the lack of generalization capability of these methods. To achieve higher detection performance for cracks, a fully convolutional network (FCN) method is used to detect cracks at the pixel level. Yang et al. [38] proposed an FCN by feeding multiple types of cracks to semantically identify and segment pixel-wise cracks at different scales. Dung et al. [39] proposed a crack detection method using VGG16-based encoder to extract feature, achieving about 90% in average precision. Bang et al. [40] proposed a pixel-level detection method for identifying road cracks in black-box images using a deep convolutional encoder–decoder network. The encoder consists of convolutional layers of the residual network for extracting crack features, and the decoder consists of deconvolutional layers for localizing the cracks in an input image. Li et al. [41] proposed a damage detection method based on the FCN to detect four concrete damages: cracks, spalling, efflorescence, and holes. This method can indeed detect multiple concrete damages at the pixel level in realistic situations. Various deep learning methods have been applied to crack detection infrastructures, but successful application of these methods for detection crack on dam surface has been rarely reported. The crack image of a dam surface has disadvantages such as large noise, complex background texture, random location of cracks, etc. To overcome these problems, we propose a pixel-level dam surface crack detection method using a deep convolutional network to extract features. Using the positioning characteristics of shallow convolution layer and the abstract features of deep convolution layer, multi-scale convolution cascade fusion and multi-dimensional loss value calculation are performed to achieve pixel-level segmentation of crack defects, and solve the problem of apparent crack detection on the dam surface with advantages such as high accuracy and high efficiency, eliminate possible safety hazards, and ensure the safety of the dam surface. The experimental results show that the proposed method is optimal for detecting pixel-level crack on dam surfaces.

## 2. Methodology

Semantic segmentation is one of the important research directions in the field of object detection. It can achieve pixel-level target detection, and has made breakthroughs in scene understanding and medical disease detection. For example, networks such as PSPNet [42], ICNet [43], Deeplabv3 [44], UNet [45] and SegNet [46] show good performance in some open datasets. In general, the semantic segmentation network consists of encoding network and decoding network [47]. In an encoding network, convolutional layers are applied to extract features of the input image. Pool layers are used to reduce the size of the feature map and the computational burden of network has also been decreased. In the decoding network, deconvolutional layers are used to restore the feature map to the size of the input image and output the prediction result. To make use of both the sparse and dense feature map, the structure of encoding network and decoding network is highly symmetrical. The location feature of the shallow layer and the abstract feature of the deep layer are fused by cascade operation. The network can integrate multi-level features and improve performance by this connecting method. In this paper, the symmetrical architecture of the network is used to detect cracks on the dam surface. Crack pixels and background pixels are segmented from input image.

**Figure 1:**
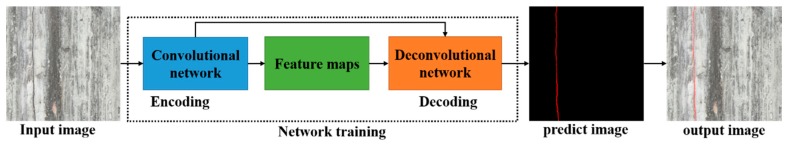
The diagram of crack detection network.

As shown in Figure 1, the schematic diagram of crack detection network. In our method, we design a pixel-level CDDS network to detect crack on a dam surface. To integrate sparse and dense features, we use a skip module to connect the encoding network and decoding network, and then calculate the loss of each skip module to improve accuracy. Next, the structure of CDDS is introduced in detail.

### 2.1. The Architecture of CDDS Network

**Figure 2:**
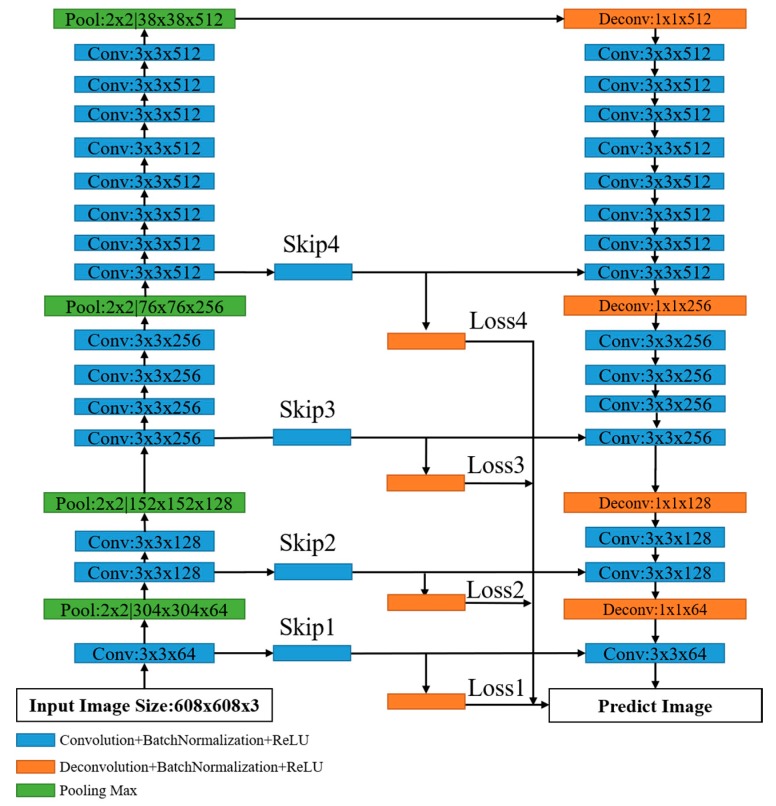
Details of crack detection on dam surface (CDDS) network structure.

The architecture of CDDS network is shown in Figure 2. The entire CDDS network consists of encoding and decoding parts, which is an improved structure combining the advantages of SegNet. The encoding part consists of 15 convolutional layers and 4 pooling layers. The decoding part consists of 15 convolutional layers and 4 deconvolutional layers. Both the encoding and decoding parts include drop layers and batch normalization layers by default. A convolution layer with kernel size of 3 × 3 and stride of 1 is used to extract features of the input image. A pooling layer with kernel size of 2 × 2 and stride of 2 is used to decrease the size of feature map, thereby reducing the computational burden. In each deconvolutional layer, the kernel size is 1 × 1, the size of output depends on the step size. The selection of kernel size is inspired by VGGNet [20] which can reduce the training parameters and thus improve the network’s operating efficiency. Configuration of the convolutional layer of the decoding part is the same as that of the convolutional layer of the encoding part. Between the encoding part and the decoding part, four skip branches are derived from different convolutional layers of the encoding network, the skip is inspired by DeepCrack [47]. Each skip branch is followed by a 1 × 1 convolutional operation and a deconvolutional operation. The result of the convolutional operation is added to the corresponding convolutional layer in the decoding part. The deconvolutional result of each branch is used to calculate a branch loss, and final the four losses are cascaded as part of total loss. The deconvolutional output size of each skip branch is equal to the size of the input image.

### 2.2. Convolutional Layer

**Figure 3:**
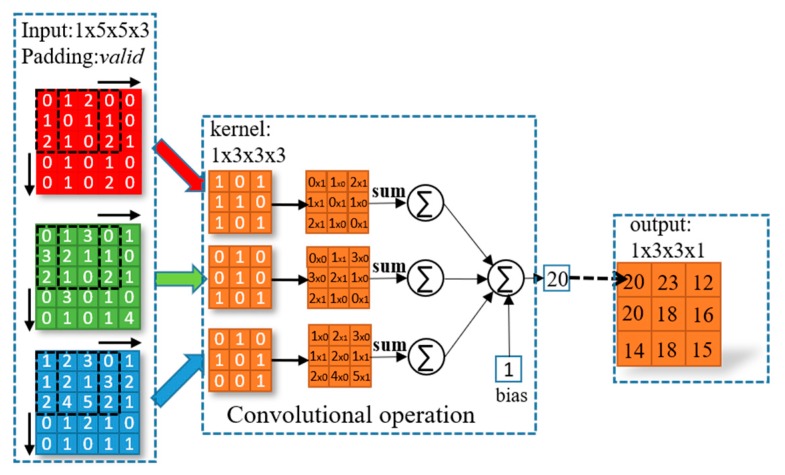
The principle of convolutional operation.

The convolution is a basic operation for extracting image features in the field of deep learning. In general, the convolution is performed on input images using a convolutional kernel with learnable parameters and a fixed stride. Convolutional operations are essentially multiplication and addition operations. The number of channels of the convolution kernel must be equal to the number of channels of the input tensor. The number of channels of the output tensor can be changed by altering the number of convolutional kernels. Each convolutional layer is followed by a corresponding bias, batch normalization and activation function. In the convolutional layer, a nonlinear activation function is used for nonlinear transformations. The CDDS network is trained by iteratively updating weight parameters and bias parameters. As shown in Figure 3, the input image is a 4-dimensional tensor with a size of 1 × 5 × 5 × 3, convolutional kernel size is 1 × 3 × 3 × 3, and final output tensor size is 1 × 3 × 3 × 1. It is worth noting that the output size of the feature map is determined by the size of convolution kernel, padding method, and stride. Usually, if the same mode is selected, it means that the convolutional output resolution is equal to the input tensor resolution. The calculation formula is as follows:

(1) $$outputsize=\{\begin{array}{cc} \frac{inputsize}{stride} & same\\ \frac{inputsize-filtersize+1}{stride} & valid \end{array}$$

where the *outputsize* is the output size, *inputsize* is the input size, stride is the step, *filtersize* is the convolution kernel size, the same means zero-padding, and valid means non-padding.

### 2.3. Pooling Layer

**Figure 4:**
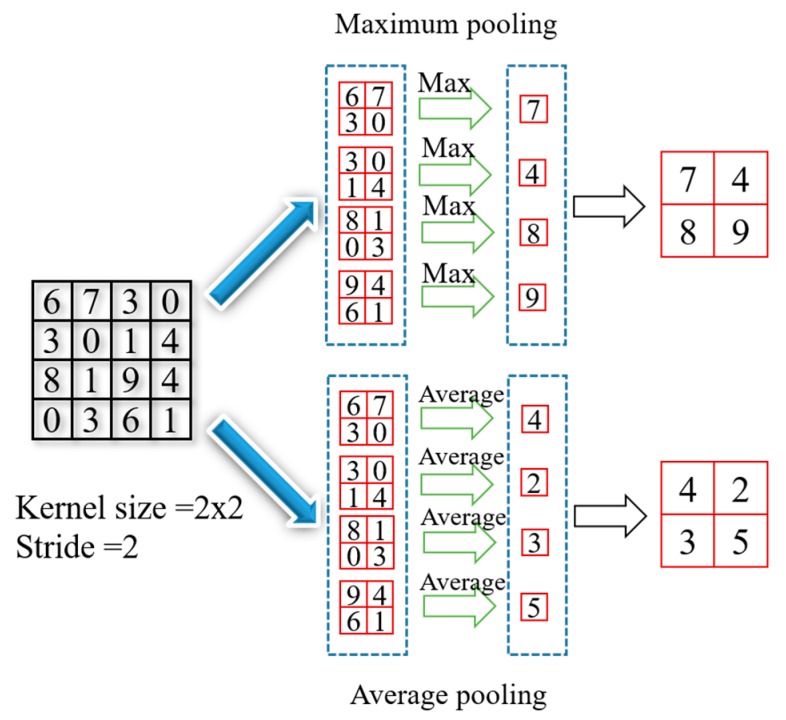
The principle of pooling operation.

Pooling is a basic operation for down-sampling in the field of deep learning. By reducing resolution of feature map, the calculation amount of network is decreased. Pooling operation includes two calculation methods: maximum pooling and average pooling. The maximum pooling uses a sliding window without weights to slide on the input tensor, and retains the maximum value in the sliding window as the output. The maximum pooling has the characteristics of translation invariance, which can adapt to the situation that the object has a certain displacement. The average pooling also uses a sliding window without weights to slide on the input tensor, and keeps the average of all values in the sliding window as the output. Figure 4 shows an example of the principle of pooling operation, the kernel size is 2 × 2, the stride is 2.

### 2.4. Deconvolutional Layer

**Figure 5:**
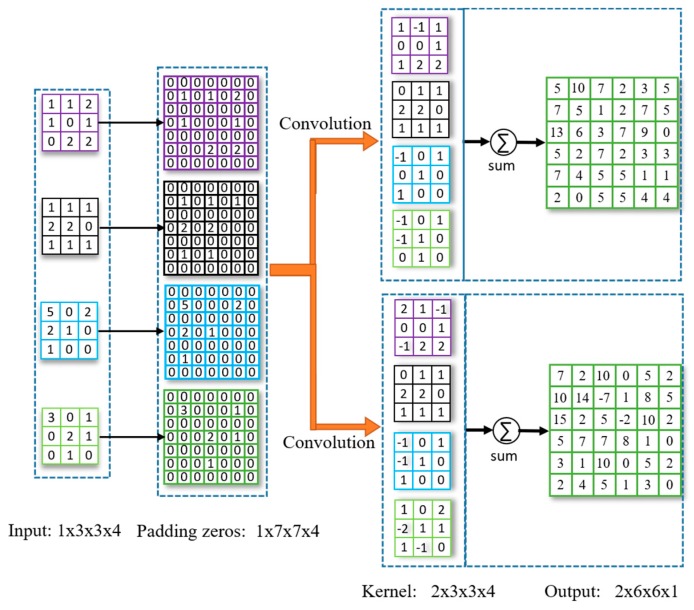
The principle of deconvolutional operation.

Deconvolution is also called transposed convolution, which is a basic up-sampling operation in the deep convolutional network. To achieve end-to-end pixel-level prediction, the size of the feature map is restored to the raw input image size by up-sampling. In general, the main methods of up-sampling include deconvolution and interpolation. Deconvolution is a simple and reliable up-sampling method that can transform small sparse matrices to large dense matrices. The calculation method of deconvolution under different deep learning frameworks is slightly different. Take the TensorFlow framework as an example, first the outermost layer of the input tensor is filled with zeros, then the output tensor is calculated using a deconvolution kernel, and the last column and last row of the output tensor are cropped. In summary, the principle of the deconvolution operation is similar to that of the convolutional operation. The calculation process of deconvolution includes convolution, padding and cropping. Figure 5 shows an example of deconvolution operation, where the size of the input tensor is 1 × 3 × 3 × 4, the size of the deconvolution kernel is 2 × 3 × 3 × 4, and the size of the output tensor is 2 × 6 × 6 × 1.

## 3. Experiments

### 3.1. Crack Database of the Dam Surface

**Figure 6:**
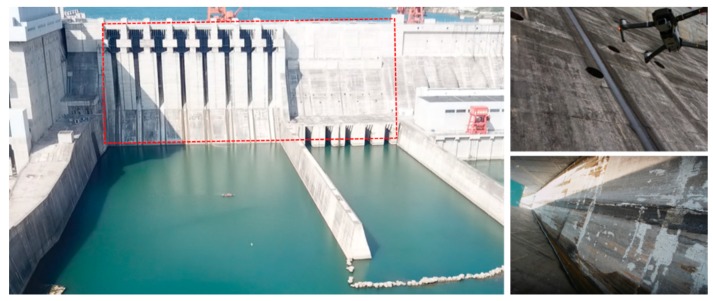
A dam of hydropower station in Jialing River Basin.

Dam images come from a dam of hydropower on the Jialing River, a tributary of the Yangtze River. The manual collection method has the disadvantages of high labor costs, high security risks and low efficiency. To quickly and efficiently establish the dam crack database, we use DJI MAVIC 2 professional UAV to collect images on the dam surface. The UAV is equipped with a 20-megapixel high-definition camera and inspects the entire dam surface along the scheduled route. Throughout the process, we control the UAV to maintain a fixed flight distance from the dam, and then make the camera lens as parallel as possible to the dam surface. Finally, 1000 raw images with a resolution of 5472 × 3648 were obtained. Figure 6 shows a dam of a hydropower station on the Jialing River Basin.

Table 1 shows the hardware equipment parameters and environmental parameters for image collection on the dam surface.

**Figure 7:**
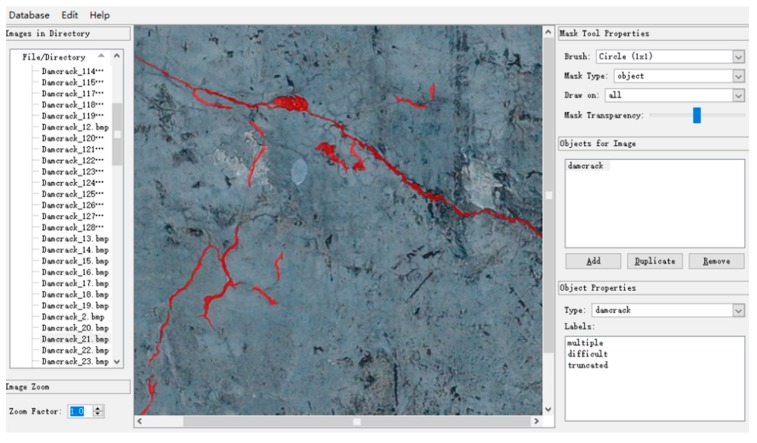
Labeling crack pixels using LEAR software.

To obtain effective images containing cracks, reduce the requirements for computer hardware performance, and shorten the training time of the CDDS network, the raw images were cropped, and flipped, and finally 504 images with a resolution of 608 × 608 were obtained. Each image contains 369,664 pixel samples, and the entire database contains 186,310,656 pixel samples. The ground truths of the collected images are manually labeled at the pixel level using the LEAR software. The ground truth of all the images was labeled by three laboratory colleagues with extensive labeling experience. Professionals formulate labeling rules, that is, which pixels in the image are crack pixels and which pixels are background pixels. To get accurate crack labels, it takes about 15 min to label an image. All images and labels in the database are RGB three-channel PNG format. In the label image, black is used to represent the background, and red is used to represent the cracks, that is, (0 0 0) represents the background pixel and (255 0 0) represents the crack pixel. Figure 7 shows the labeling of crack pixels using LEAR software.

**Figure 8:**
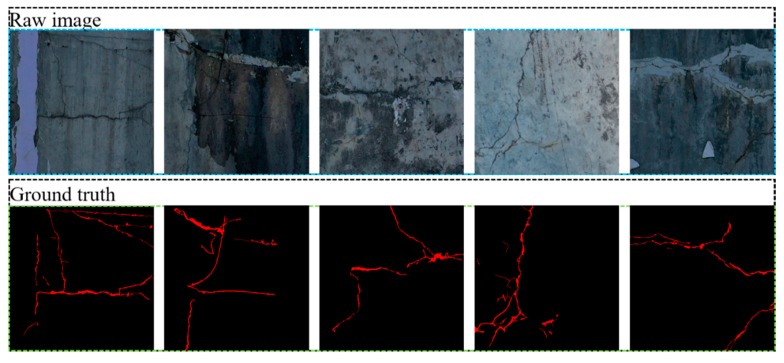
The example of crack images and ground truths for dam surface.

To train the CDDS network and verify its performance, 80% of the samples in the database are used for training, 10% of the samples are used for verification, and 10% of the samples are used for testing. A total of 504 images in the database are randomly shuffled, 404 images are divided into training dataset, 50 images are divided into the validation dataset, and the last 50 images are used as the test dataset. Cracks on the dam surface have the disadvantages of patching, noisy images, complex textures, unstructures, uneven distribution, and blurred backgrounds. These disadvantages bring many challenges to crack detection on a dam surface. Figure 8 shows an example of crack images and ground truths on the dam surface.

### 3.2. Experimental Settings

TensorFlow is an open source deep learning framework. The architecture of the CDDS network is built using TensorFlow on a Linux system. Training, verifying, and testing are performed on a HP workstation configured with 8 GB GPU. The computational task of image is very suitable for GPU. Anaconda is a software of environment creation; it can be used to create different python environments for different scenarios. A virtual python environment for CDDS network is established using Anaconda. CUDA and CUDNN are calculation libraries used to speed up GPU, thereby improving the training speed of the network. The software version and hardware parameters of the workstation are shown in Table 2.

Each pixel is classified instead of the entire image for enabling pixel-level detection. The CDDS network is trained with a batch size of one image, a momentum of 0.99, and a weight delay of 0.005 for 100 epochs. To verify initial learning rate, our training processes use the value of 10^−4^, 10^−5^, 10^−6^. As the depth of deep convolutional network continues to increase, overfitting is prone to occur when the number of training samples is insufficient. Dropout can reduce the complexity of the network and improve its performance. Therefore, the CDDS network uses the dropout rate of 0.2. The rectified linear unit (ReLU) was introduced [48] as a nonlinear activation function. It does not have the problem of gradient disappearance because the gradients of the ReLU are always zero and one. Root Mean Square Prop (RMSProp) is an optimization algorithm that optimizes the problem of excessive swing amplitude of the loss function in the update, and further accelerates the convergence speed of the function. We select ReLU as the activation function and RMSProp as the optimizer in the experiment. Every epoch, 30 images are randomly selected from the verification dataset to verify the performance of current network. It takes about 9 h to complete training for the entire network. To make the network focusing on crack samples, diceloss function is used to calculate the loss value of the network. The formula is as follows.

(2) $$loss=\frac{2\times true\cap pred}{true\cup pred+true\cap pred}$$

where *true* represents the ground truth of input image, *pred* represents prediction result of the network.

### 3.3. Evaluation Metrics of the Network

Pixel-level crack detection is essentially binary classification for each pixel. Precision, recall and F-measure are classic evaluation indicators of binary classification. Therefore, these indicators can be used to assess the performance of crack detection.

The precision represents the probability that the ground truth of the sample is also cracked in all samples which were predicted to be cracked. The recall indicates the probability of sample being predicted as cracked in all samples labeled as cracked. When there is a large gap between the number of positive and negative samples, it is not reasonable to use only precision or recall to evaluate performance. Considering the combined effect of recall and precision, F-measure is a comprehensive indicator. IoU is commonly used in the field of object detection to evaluate locating accuracy. The IoU represents the ratio of the intersection of the predicted result and the ground truth to that of union. The crack image of the dam surface contains two categories of background and crack. The number of background pixels is much larger than the number of crack pixels. Usually, we calculate the IoU of background and the IoU of crack at the same time, and then use the average of the two IoUs as the final IoU. The value of IoU is affected by the background pixels and cannot accurately express the locating accuracy of the crack. It is more appropriate to use the IoU of the crack as a locating indicator in our paper. The calculation formula for the evaluation indicators are as follows.

(3) $$precision=\frac{TP}{\left(TP+FP\right)}$$

(4) $$TPR=recall=\frac{TP}{\left(TP+FN\right)}$$

(5) $$TNR=\frac{TN}{\left(TN+FP\right)}$$

(6) $$F-measure=\frac{2\times recall\times precision}{\left(recall+precision\right)}$$

(7) $$IoU_{crack}=\frac{TP}{TP+FN+FP}$$

(8) $$IoU_{background}=\frac{TN}{TN+FN+FP}$$

where *TP* indicates that the ground truth is a crack pixel and the prediction result is also a crack pixel; *FP* indicates that ground truth is a *background* pixel, but prediction result is a *crack* pixel; *FN* indicates that ground truth is a *crack* pixel, but prediction result is a *background* pixel; *TN* indicates that ground truth is a *background* pixel, and prediction result is also a *background* pixel. *TPR* represents the probability of being correctly predicted as a *crack* in all samples labeled as cracks. *TNR* represents the probability of being correctly predicted as the *background* in all samples with the label as the *background*. $IoU_{crack}$ represents the *IoU* of *crack*. $IoU_{background}$ represents the *IoU* of *background*. A more intuitive expression is shown in Table 3.

## 4. Results and Discussion

### 4.1. Results of CDDS Training

After the hyper-parameters are configured, the training of network parameters is launched. The training dataset has 404 images, and one input image is used to train during each iteration, so that each epoch requires 404 iterations to traverse the entire training dataset. At every iteration, the training loss of the current network is calculated. After each epoch, the network weights are saved. The learning rate has a certain effect on the network’s convergence speed, the network is trained with 3 different learning rates. All images contain background and crack pixels, the output size of the CDDS network is 608 × 608 × 2. The softmax function is used to obtain a probability, and then the dice-loss function is applied to calculate the loss value of network. After the network training is completed, the precision, recall, F-measure and IoU are calculated on the test dataset to assess performance of the network.

**Figure 9:**
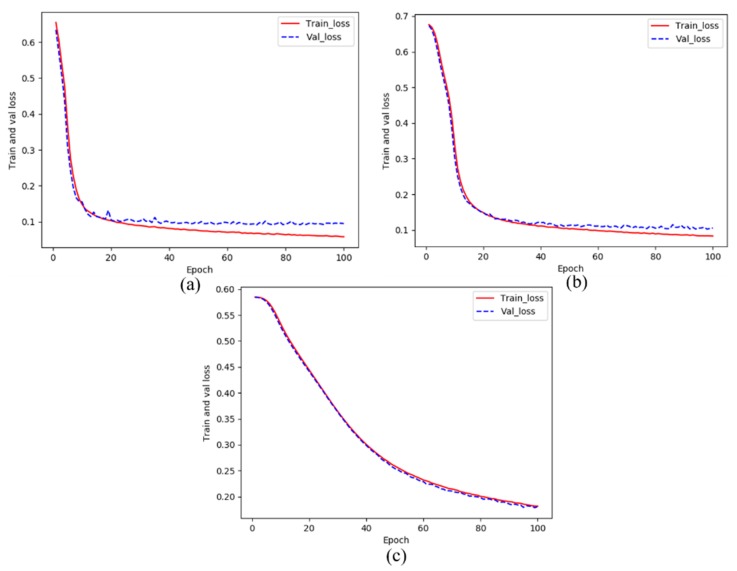
The training losses and validating losses over training epoch: (**a**) learning rate = 10^−4^, (**b**) learning rate = 10^−5^, (**c**) learning rate = 10^−6^.

Figure 9 shows the loss curves of training and validating during the training of CDDS network. The solid red line represents the loss curve in the training dataset, and the blue dotted line indicates the loss curves in the verification dataset. To verify a proper initial learning rate, this experiment process checked the values of 10^−4^, 10^−5^ and 10^−6^. It can be seen from the figure that the convergence rate is the fastest with a learning rate of 10^−4^, although there is a gap between validating loss and training loss, the gap is within the appropriate range. The gap between training loss and validating loss in (b) is smaller than that of (a). The learning rate 10^−5^ does not perform as well as the learning rate 10^−4^ in the test dataset, which may be caused by overfitting. The convergence rate of (c) is slow, the downward trend is relatively stable, and the training loss curve is almost consistent with the validating loss curve.

**Figure 10:**
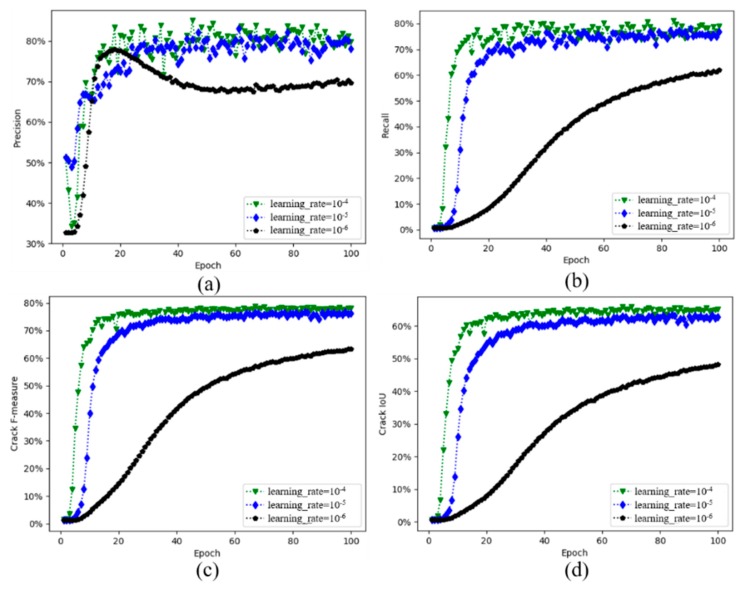
Evaluation indicators on validation dataset: (**a**) Precision, (**b**) Recall, (**c**) F-measure, (**d**) Crack IoU.

Figure 10 shows the curve of evaluation indicators on the validation dataset over training process. These evaluation indicators include: precision, recall, F-measure and crack IoU. Each indicator curve is different at three different initial learning rates. Before the 20th epoch, the precision curve rises rapidly. Whether it is the recall, F-measure or crack IoU, the trend of the black curve with the learning rate of 10^−6^ is relatively smooth. However, the convergence of the learning rate 10^−6^ takes a long time, and it is more prone to overfitting. Although the precision curve with a learning rate of 10^−4^ fluctuates significantly in the early stage, from the change trend of the recall rate, F-measure and IoU curves, the CDDS network with the learning rate of 10^−4^ has a good convergence speed and performance.

**Figure 11:**
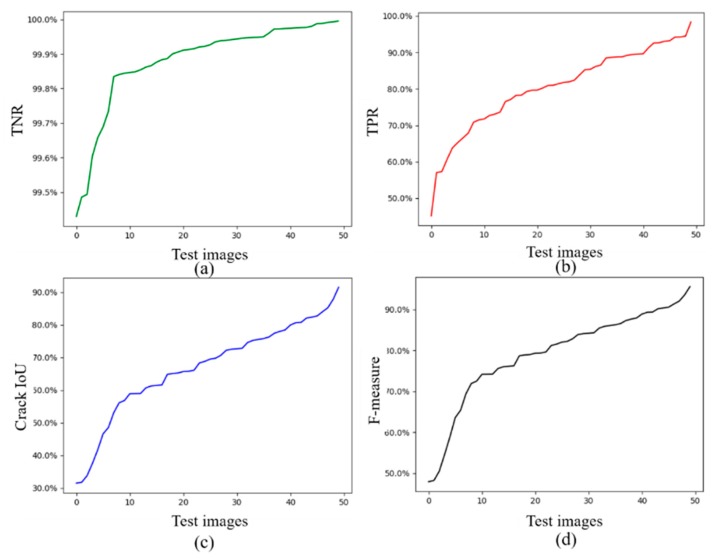
Indicator results of 50 testing images. (**a**) TNR, (**b**) TPR, (**c**) Crack IoU, (**d**) F-measure.

Figure 11 shows the evaluation results of the CDDS network on the test data set, the learning rate is 10^−4^. There are 50 images in the test dataset. The TNR, the TPR, the F-measure and the crack IoU are calculated for each image. The evaluation indicators in the figure are arranged in ascending order of each indicator. The trend of the F-measure line and the crack IoU curve are similar. The TNR curve has a high amplitude, indicating that the recognition accuracy of the background pixels is high. The highest value of each indicator exceeds 80%.

### 4.2. Calculation of Crack Size on the Dam Surface

**Figure 12:**
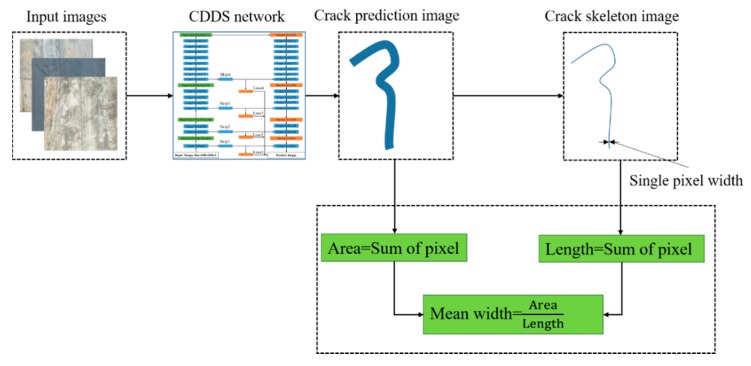
Schematic diagram of the size of the crack calculation process.

To more conveniently calculate the length, width, and area of the crack, skeleton extraction is required for the crack. The result of skeleton extraction is to use a crack with a single pixel width to represent the original crack. A commonly used skeleton extraction algorithm is a fast refinement algorithm [49]. The skeleton is quickly extracted by calling the opencv library. When the width of the skeleton is the width of a single pixel, the length of crack can be obtained by summing the skeleton pixels, the area is the sum of crack pixels, and the average width is obtained by the ratio of the area to the length. A brief description of the entire crack size calculation process is shown in Figure 12.

(9) $$S_{crack}=\sum_{\;i,j\;\in \;C_{c}}^{}p_{ij}$$

(10) $$L_{crack}=\sum_{\;m,n\;\in \;C_{l}}^{}l_{mn}$$

where $S_{crack}$ represents the area of the crack, $p_{ij}$ represents the pixels of the crack, $C_{c}$ represents the set of the crack pixels; $L_{crack}$ represents the length of the crack; $l_{mn}$ represents the pixels of the skeleton; $C_{l}$ represents the set of the skeleton pixel.

**Figure 13:**
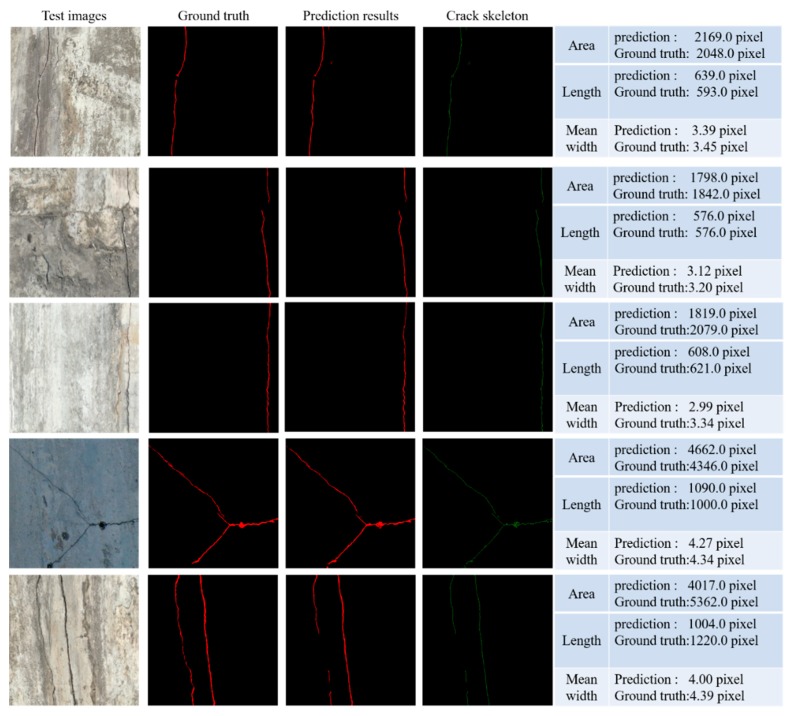
Examples of crack skeleton extraction and size calculation.

Figure 13 shows examples of crack skeleton extraction and size calculation. The width of crack skeleton is expressed by a single pixel. The length of the crack is calculated from the crack skeleton, and the actual crack size is calculated from the ground truth. It can be seen from the results that the calculation error of the length and average width is small, and the error of the prediction of the area of the crack is relatively large. In the entire test dataset, the variation range of the relative error of the crack area is from −35.02% to 119.94%; the variation range of the relative error of the crack length is from −35.12% to 73.13%; and the variation range of the relative error of the mean width is from −32.84% to 58.69%.

**Figure 14:**
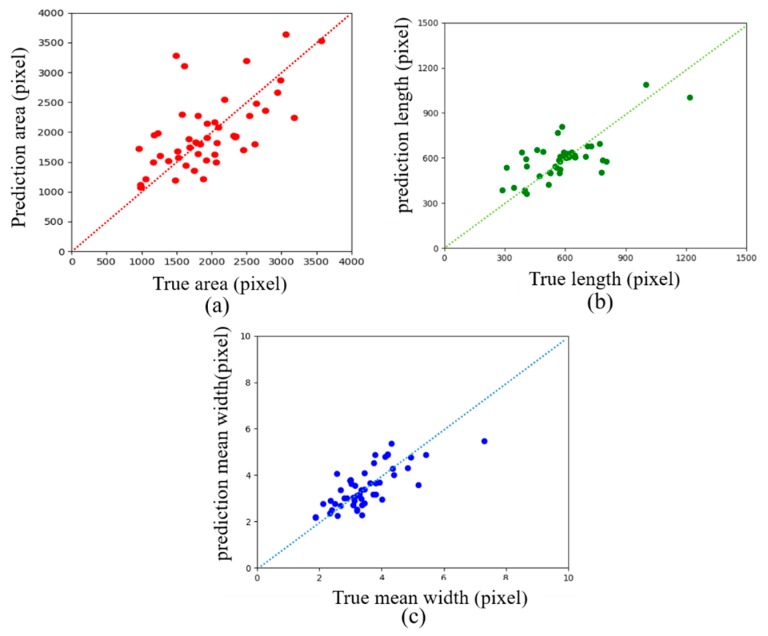
Statistics of crack size in test dataset: (**a**) crack area, (**b**) crack length, (**c**) crack mean width.

Figure 14 shows the comparison of the predicted value and the ground truth of crack size in the test dataset. Most data points of the mean width are located near y = x, with small deviations. The data points of area and length have relatively large deviations.

**Figure 15:**
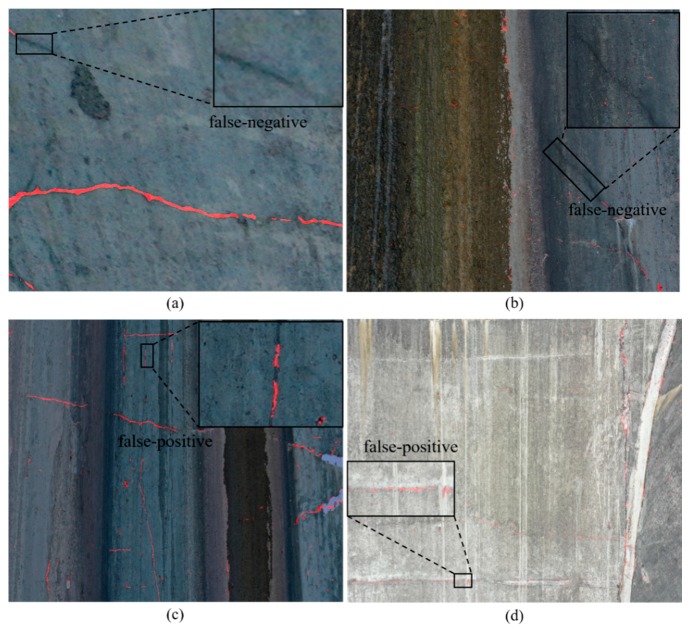
Examples of errors in the test results: (**a**) and (**b**) show false-negative errors; (**c**) and (**d**) show false-positive errors.

Figure 15 shows some examples of error in the test results of the proposed methods. (a) and (b) show false-negative errors, which means that the ground truth is crack but the prediction is background. (c) and (d) show false-positive error, which means that the ground truth is background but the prediction is crack.

## 5. Comparative Study

**Figure 16:**
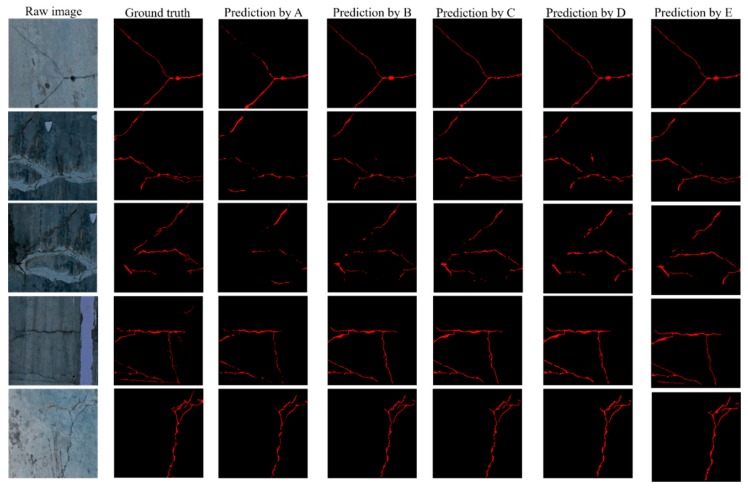
Examples of comparison of prediction results. (A represents FCN, B represents UNet, C represents SegNet, D represents ResNet152-based and E represents the proposed method).

To verify the pixel-level segmentation performance of the proposed method, this paper uses three network models for comparative research: UNet [45], SegNet [46], FCN [38] and ResNet152-based [40]. UNet is a typical network in the field of medical image segmentation, and the structure of UNet is very symmetrical. SegNet is a deep convolutional encoding and decoding network architecture. The encoding part of the network is composed of the convolution of the VGG16 model, and the segmentation performance in the street view dataset is good. FCN is a modified network architecture for concrete crack detection based on VGG19. The road crack of the ResNet152-based is a pixel-level road crack method, the decoding layer of this method uses ResNet152 network to extract image features. The learning rate of 0.0001 is best for the ResNet152-based network; UNet and SegNet use a momentum of 0.995 and the RMSprop is selected as the optimizer. The learning rate of 0.001 is best for the SegNet network; the learning rate of 0.0001 is suit for UNet. The best learning rate for FCN is 0.00001. These parameters are selected over by many experiments. The experimental comparison results are shown in Figure 16.

Figure 16 shows some examples of prediction results for the ResNet152-based, SegNet, Unet, FCN and CDDS method in test dataset. Different methods perform differently in the details of crack prediction results. Table 4 shows the comparison results of the proposed CDDS network and other methods in the test dataset. The indicators of the proposed CDDS network are higher than other methods. The number of background pixels in the entire dataset is much higher than the crack pixels. To express the location performance of the crack more accurately, the IoU of crack and background are calculated respectively. The background IoU of each method is above 90%. The crack IOU and the F-measure for the proposed CDDS network reach 66.76% and 79.16%, respectively. Table 5 shows the comparison of training and testing time for the proposed CDDS network and other methods in the test dataset. Training time includes image reading time, network training and network verification. Test time indicates the average test time of each picture.

**Figure 17:**
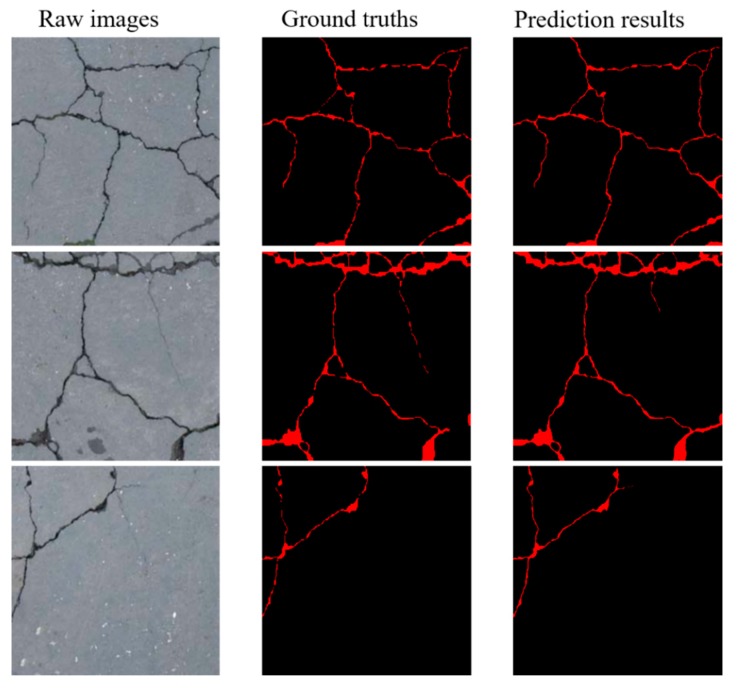
Examples of prediction results using proposed method on the road crack dataset.

To validate the performance of our proposed method on other public crack datasets we collected 764 images of road cracks from the Internet. These road crack images have corresponding pixel-level labels. This crack dataset is also divided into a training dataset, a validation dataset, and a test dataset. The training dataset contains 620 images, and the validation and test datasets each contain 72 images. If all the hyper-parameters are kept constant, such as learning rate, batch size, we retrained our proposed method on the training dataset. As shown in Figure 17, the test results on the test dataset show the effectiveness of the proposed method for the pixel-level detecting cracks. On the test dataset, the crack IOU and the F-measure for the proposed CDDS network reach 67.41% and 80.14%, respectively.

## 6. Conclusions

A pixel-level crack detection method using a deep convolutional network is proposed to detect crack on dam surface. The CDDS network is improved based on the characteristics of the SegNet structure and consists of encoding and decoding parts. The encoding part is used to extract the feature of the input image, the decoding part is used to output the pixel-level prediction results. In the architecture of CDDS, four skip branches are used for combining the shallow and deep features of the network. Each skip branch is followed by a convolution layer and a deconvolution layer. The losses of the 4 skip layers are added as part of the total loss. A UAV with a high definition camera was used to collect 1000 raw images with a resolution of 5472 × 3648. To augment the dataset, 504 valid images with a resolution of 608 × 608 obtained by cropping and flipping. The entire dataset is divided into three subsets, of which the training dataset has 404 images, the test dataset has 50 images, and the validation dataset has 50 images. After the CDDS network training, to obtain the size of the crack, we need to extract the skeleton of the crack. The sum of skeleton pixels represents the length of the crack. The sum of crack pixels in the prediction results indicates the area of the crack. The average width of the crack is calculated using area and length. The precision, the recall, the F-measure and the IoU are calculated in the test dataset. The recall, precision, F-measure, crack IoU and background IoU the for the proposed CDDS network reach 80.45%, 80.31%, 79.16%, 66.76% and 99.76%, respectively. Compared with other pixel-level detection networks, the proposed CDDS network has higher indicators and performance. In future studies, we will continue to improve the accuracy of crack location techniques.

## Figures and Tables

**Table 1:** Dam surface image collection equipment and environmental parameters.

Hardware/Environmental	Specifications/Parameters
UAV	DJI MAVIC 2
Sensor	1’’CMOS; Effective Pixels: 20 million
Camera Lens	35 mm Format Equivalent: 28 mm
Light Condition	5000 Lux–20000 Lux
Wind Speed	1 m/s–2 m/s
Total Collection Time	18 h
Collection Distance	3 m

**Table 2:** Experimental software version and hardware configuration.

Hardware/Software	Specifications/Parameters/Version
CPU	Inter^®^Xeon(R) CPU E5-2650 v4 @ 2.20 GHz × 48
GPU	Quadro P4000/PCIe/SSE2/8 GB
RAM	62.8 GB
CUDA	9.1
CUDNN	7.1.5
Python	2.7.5
Anaconda	3–5.1.0
TensorFlow	1.10

**Table 3:** The detailed description of TP, FP, TN, FN.

Ground Truth	Prediction Results	
	Crack	Background
**Crack** **Background**	TP	FN
	FP	TN

**Table 4:** Comparison of indicator parameters for ResNet152-based, FCN, Unet, SegNet and CDDS.

Methods	Recall (%)	Precision (%)	F-measure (%)	Crack IoU (%)	Background IoU (%)
ResNet152-based	57.49	74.99	63.68	47.68	99.54
FCN	71.53	72.57	69.37	55.70	99.69
UNet	78.33	77.14	76.20	62.71	99.73
SegNet	79.15	77.85	77.22	64.37	99.74
CDDS	80.45	80.31	79.16	66.76	99.76

**Table 5:** Comparison of training and testing time for ResNet152-based, FCN, Unet, SegNet and CDDS.

Methods	Training Time	Testing Time (for Per Image)
ResNet152-based	3 h and 12 min	0.13 s
FCN	6 h and 4 min	0.17 s
UNet	9 h and 17 min	0.20 s
SegNet	6 h and 2 min	0.21 s
CDDS	9 h and 24 min	0.26 s
